# Suppression of lipopolysaccharide-induced corneal opacity by hepatocyte growth factor

**DOI:** 10.1038/s41598-021-04418-x

**Published:** 2022-01-11

**Authors:** Elsayed Elbasiony, WonKyung Cho, Sharad K. Mittal, Sunil K. Chauhan

**Affiliations:** Department of Ophthalmology, Schepens Eye Research Institute of Mass Eye and Ear, Harvard Medical School, Boston, MA USA

**Keywords:** Corneal diseases, Preclinical research

## Abstract

Keratitis induced by bacterial toxins, including lipopolysaccharide (LPS), is a major cause of corneal opacity and vision loss. Our previous study demonstrates hepatocyte growth factor (HGF) promotes epithelial wound healing following mechanical corneal injury. Here, we investigated whether HGF has the capacity to suppress infectious inflammatory corneal opacity using a new model of LPS-induced keratitis. Keratitis, induced by two intrastromal injections of LPS on day 1 and 4 in C57BL/6 mice, resulted in significant corneal opacity for up to day 10. Following keratitis induction, corneas were topically treated with 0.1% HGF or PBS thrice daily for 5 days. HGF-treated mice showed a significantly smaller area of corneal opacity compared to PBS-treated mice, thus improving corneal transparency. Moreover, HGF treatment resulted in suppression of α-SMA expression, compared to PBS treatment. HGF-treated corneas showed normalized corneal structure and reduced expression of pro-inflammatory cytokine, demonstrating that HGF restores corneal architecture and immune quiescence in corneas with LPS-induced keratitis. These findings offer novel insight into the potential application of HGF-based therapies for the prevention and treatment of infection-induced corneal opacity.

## Introduction

Microbial keratitis is a major cause of corneal opacity and loss of vision globally^1^. Common causative organisms associated with microbial keratitis are *Pseudomonas aeruginosa*, Staphylococcus aureus, and coagulase-negative staphylococci^2^ and bacterial toxins, such as lipopolysaccharide (LPS)^3,4^. Throughout the period of keratitis, infiltration of inflammatory cells is associated with opacity formation impeding the transmission of light through the cornea^5^. Despite the importance of early diagnosis and intervention to maintain optimal corneal function, no treatment is available to suppress corneal opacity formation resulting from microbial keratitis.

Hepatocyte growth factor (HGF), an endogenous protein, exerts mitogenic and morphogenic effects through tyrosine phosphorylation of its receptor, c-Met^6^. In the cornea, epithelium, keratocytes, and endothelium express HGF^7,8^. We have previously reported that HGF promotes the proliferation of corneal epithelial cells in homeostatic conditions and in the inflammatory environment, resulting in faster corneal re-epithelization following mechanical injury^9,10^. Moreover, patients with keratoconus and conditions associated with poor epithelial healing have been reported to show decreased expression of c-Met^11,12^. Despite their known proliferative function, the therapeutic efficacy and underlying role of HGF in corneal epithelial repair, in particular to fibrosis, have only been recently explored. Our group showed that HGF suppresses α-smooth muscle actin (α-SMA) expression in corneal fibroblasts to prevent fibrosis following corneal injury^13^. Moreover, HGF treatment has also been shown to induce apoptosis of corneal myofibroblasts to restore transparency following alkali injury^14^. Given the detrimental aftermath of corneal opacity following keratitis, understanding the effect of HGF administration in suppressing keratitis-induced corneal opacity will be of high clinical benefit.

In the current study, we established a new murine model of LPS-induced keratitis in which significant corneal opacity was observed following two consecutive intrastromal injections of LPS. Using this model, we investigated the therapeutic potential of HGF in suppressing the development of corneal opacity to preserve transparency in LPS keratitis. Our data demonstrate that topical HGF treatment significantly reduces the severity of inflammatory opacity to preserve corneal transparency following keratitis. Moreover, administration of HGF significantly reduces the expression of the fibrotic marker α-SMA, restores tissue architecture, and suppresses corneal inflammation.

## Results

### Multiple administration of LPS induces corneal opacity

**Figure 1:**
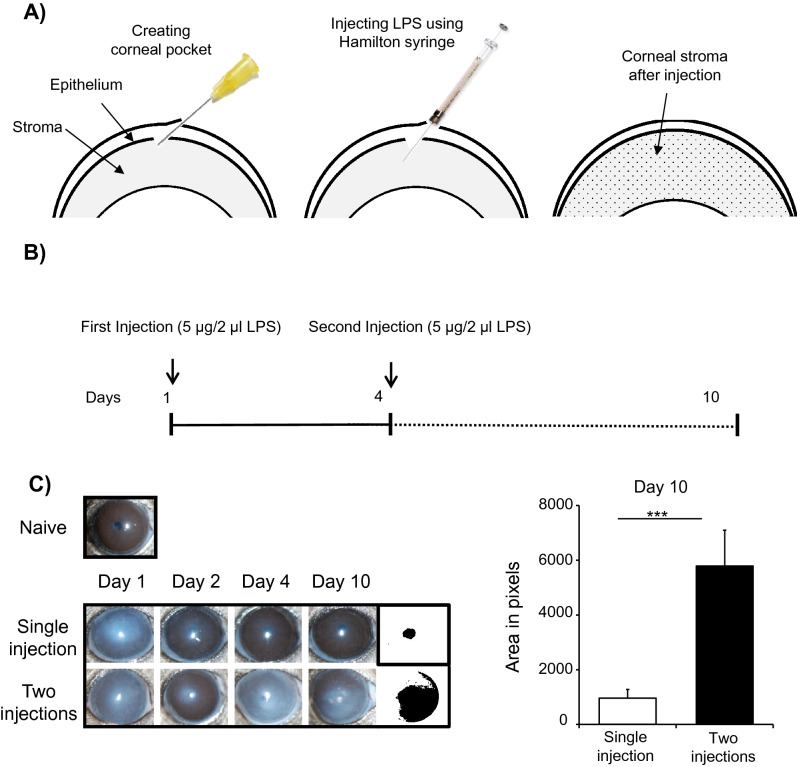
Murine model of LPS-induced corneal opacity. A corneal pocket was created using a 30 G needle and 5 µg of LPS in 2 µl of PBS were injected using a 2.5 µl Hamilton syringe at two time points (Day 1 and Day 4). (**A**) Schematic diagram depicting the procedure of LPS-induced keratitis. (**B**) Schematic of the experimental design (**C**) Representative bright-field images of corneas injected with one or two injections of LPS on day 1, 2, 4 and 10. Representative data from three independent experiments are shown, and each experiment consisted of three animals. Data presented are mean ± SEM. ***p < 0.001.

In the current study, we developed a murine model of microbial keratitis with two sequential intrastromal administrations of LPS. Through a partial thickness tunnel, 5 µg of ultrapure *Escherichia coli* LPS in 2 µl PBS were injected into the corneal stroma. Successful injection was identified by stromal overhydration and edema (Fig. 1A). Mice injected with either one or two doses of LPS were followed up for 10 days to determine the efficacy of each model in developing corneal inflammatory haze (Fig. 1B). Mice treated with two injections of LPS (Day 1 and Day 4) developed significant corneal opacity up to day 10 (Fig. 1C) (*p* = 0.0003).

### HGF penetrates corneas with LPS-induced keratitis

**Figure 2:**
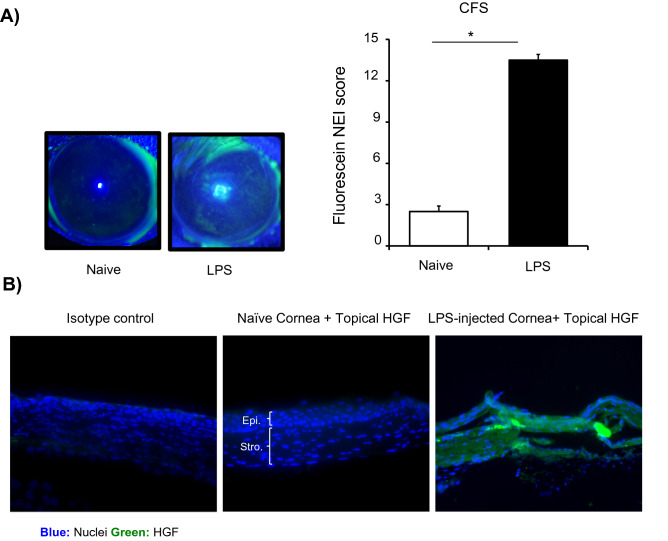
Penetrance of topically applied HGF in LPS-induced keratitis. Keratitis was induced by corneal injection of LPS on day 1 and day 4. (**A**) Representative images (Left) and cumulative bar chart (Right) quantifying corneal epitheliopathy in LPS-treated corneas, compared to naïve controls. (**B**) Following the second intrastromal injection of LPS, corneas were treated with his-tagged HGF thrice a day. Cross-sections of corneas, harvested 24 h post HGF treatment, were stained with anti-His-tag antibody (green) to evaluate the penetrance of exogenously administered HGF into the corneal stroma (Scale bars, 100 μm). Representative data from three independent experiments are shown, and each experiment consisted of three to four animals. Data presented are mean ± SEM. *p < 0.05.

Next, we sought to determine whether topically applied HGF penetrates corneal epithelium into the stroma in the new model of LPS-induced keratitis. Following the second intrastromal injection of LPS, corneal fluorescein pictures were captured and analyzed using the National Eye Institute (NEI) fluorescein scoring scale to assess corneal epitheliopathy. We observed a significant increase in corneal epitheliopathy in LPS treated corneas relative to controls, as demonstrated by punctuated green staining (Fig. 2A) (*p* = 0.03). To measure penetrance of HGF, corneas were treated with polyhistidine C-terminal tagged HGF following induction of keratitis for 24 h. Cross sections of harvested corneas were stained with anti-His-tag antibody. Diffusely distributed HGF staining throughout the corneal stroma in corneas with keratitis compared to naïve controls demonstrated that exogenously administered HGF penetrates damaged stroma with high efficacy (Fig. 2B). Taken together our data suggest that the barrier function of the corneal epithelium is disrupted in LPS-induced keratitis, which allows the stromal penetration of topically applied HGF.

### HGF penetrates reduces keratitis-induced corneal opacity

**Figure 3:**
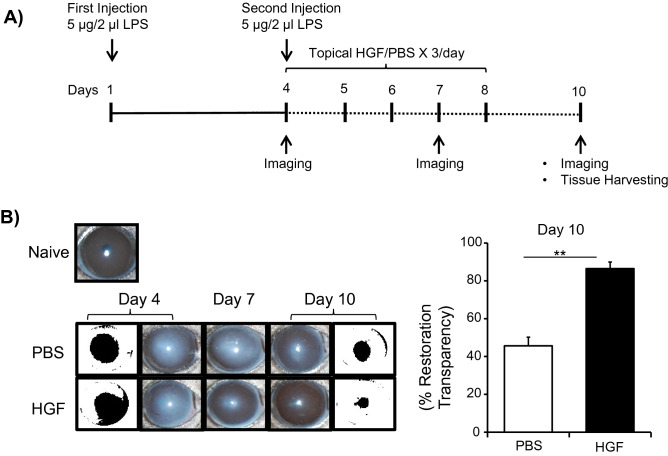
Topical administration of HGF prevents keratitis-induced corneal opacity. Keratitis was induced by corneal injection of LPS on day 1 and day 4 followed by topical application of 2 µl of 0.1% HGF thrice a day for five days. Bright-field images were captured on days 4, 7, and 10 following the first injection. (**A**) Schematic of experimental design, (**B**) Representative bright-field images (left) cumulative bar chart (right) depicting the size of corneal opacity in mice treated with the indicated treatment. Bright-field images were converted into a binary mode (black and white) using ImageJ software with black corresponding to high opacity areas. Results were expressed as percent restoration of corneal transparency on day 10 compared to day 4. Representative data from experiments consisting of 6 animals per treatment group. Data presented are mean ± SEM. **p < 0.01.

To determine the effect of HGF on corneal opacity following keratitis, mice were divided into two groups following the second intrastromal injection of LPS. Group 1 was topically treated with 3 µl of HGF using a micropipette and group 2 with PBS as outlined in the experimental design (Fig. 3A). Brightfield images were captured on the day of treatment initiation (Day 4) and treatment completion (Day 10) (Fig. 3B). Using NIH Image J software, images were converted into a binary mode (black and white) and the size of the black area, representing corneal opacity, was calculated. We noticed a significant restoration of corneal transparency in HGF treated mice (two-fold; *p* = 0.008) as indicated by the smaller size of corneal opacity compared to the PBS-treated mice. These data demonstrate that HGF effectively preserves transparency and prevents corneal opacity following microbial keratitis.

### HGF reduces keratitis-induced corneal fibrosis

**Figure 4:**
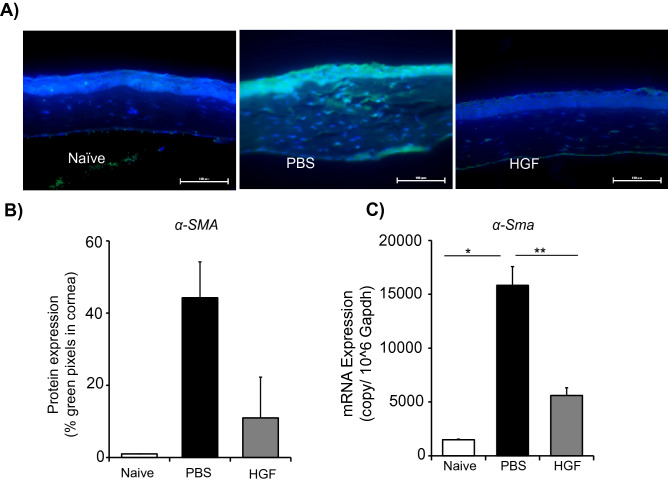
HGF reduces the expression of fibrotic marker α-SMA. Corneas were harvested from mice treated with PBS or HGF on day 10 post-injection. (**A**) Corneal cross-sections were prepared and immunostained with α-SMA (green) to evaluate corneal fibrosis (Scale bars, 100 μm). (**B**) Cumulative bar chart depicting protein expression (% green pixels in the cornea) in indicated groups. Data presented are mean ± SEM (error bar). (**C**) Bar chart depicting expression of fibrotic marker α-SMA in the cornea, as quantified by real-time PCR. Representative data from experiments consisting of 6 animals per treatment group. Data presented are mean ± SEM. *p < 0.05, **p < 0.01.

To confirm the efficacy of HGF in reducing corneal opacity formation and preserving transparency following LPS keratitis, we investigated whether HGF inhibits the expression of α-smooth muscle actin (α-SMA), a marker of fibrosis, following LPS injection. Eyeballs were harvested on day 10, and cross-sections stained with anti-α-SMA and DAPI were analyzed under a fluorescent microscope (Fig. 4A). A notable decrease in infiltration of immune cells, marked by reduced number of stromal DAPI-stained nuclei, was observed in HGF treated corneas compared to PBS control. HGF treatment resulted in ~ 50% suppression of α-SMA expression compared to PBS treatment (Fig. 4B). Furthermore, a significant decrease in the expression of α-SMA at the mRNA level was observed in HGF treated mice (*p* = 0.01) relative to PBS-treated control (Fig. 4C). These data demonstrate the functional capacity of HGF in reducing the expression of fibrotic marker α-SMA at both the mRNA and protein levels in LPS keratitis.

### HGF normalizes tissue structure and reestablishes immune quiescence post-keratitis

**Figure 5:**
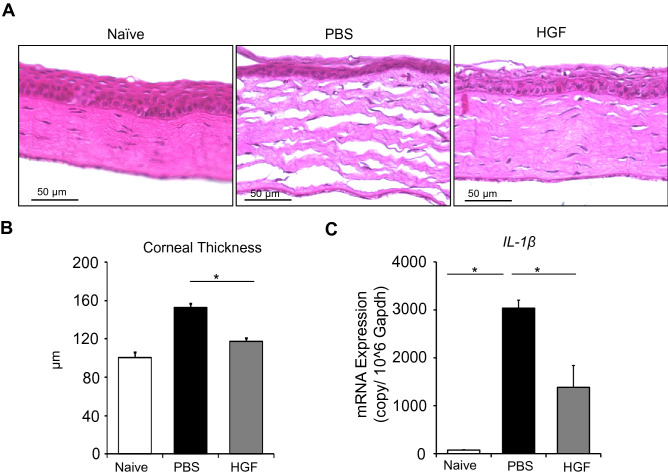
HGF normalizes corneal tissue architecture and restores immune quiescence. Corneas were harvested from mice treated with PBS or HGF on day 10 post-injection. (**A**) Cross-sections were stained with H&E to visualize corneal tissue structure. (**B**) Corneal tissue thickness was measured using ImageJ software (Scale bars, 50 μm). (**C**) Expression of pro-inflammatory cytokine IL-1β in corneal lysates was quantified by real-time PCR. Representative data from experiments consisting of 6 animals per treatment group. Data presented are mean ± SEM. *p < 0.05.

As the transparency of the cornea is required for light transmission is dependent on its uniquely organized cellular and collagen architecture, we investigated the effect of HGF in preserving normal cellular architecture and corneal thickness following LPS keratitis. Corneas were harvested on day 10 following thrice daily HGF or PBS treatment for five days and cross-sections were stained with H&E to evaluate tissue structure. HGF-treated corneas showed comparable corneal architecture and thickness (≈ 120 µm) to naive corneas (≈ 85 µm), whereas PBS-treated corneas showed a significant stromal thickness increase (≈ 160 µm) (Fig. 5A, B).

Inflammation, specifically cytokine IL-1β, has been shown to be a critical component of the fibrotic pathway^15,16^. Thus to study the effect of HGF on mediating corneal inflammation, corneas were harvested on day 10 following the first injection and mRNA expression of pro-inflammatory cytokine IL-1β was analyzed using quantitative real-time PCR. Along with the infiltration of immune cells (Fig. 5A), we observed a significant increase in the mRNA expression of IL-1β in the PBS-treated mice (*p* = 0.03) compared to naïve controls. In contrast, HGF treated mice showed a significant reduction in the expression of IL-1β, compared to PBS treated group (*p* = 0.03) (Fig. 5C). Taken together, these results indicate that HGF treatment suppresses corneal infiltration of inflammatory cells and their expression of pro-inflammatory cytokine IL-1β during LPS keratitis.

## Discussion

Microbial keratitis, a major global cause of blindness due to corneal scarring, poses a significant health problem^17^. Although the current broad-spectrum anti-microbial regimen is effective in treating bacterial keratitis, the outcomes remain poor due to the secondary corneal opacity and scarring^18^. In this study, we developed and utilized a novel murine model of corneal opacity formation following LPS-induced keratitis to test the effect of topical HGF administration on secondary corneal opacity. Our data demonstrate that topical HGF administration reduces the development of corneal opacity by suppressing the expression of pro-fibrotic marker α-SMA in the cornea, improving corneal transparency following keratitis compared to PBS control. In addition, HGF treatment normalized tissue architecture and reduced corneal inflammation.

The corneal epithelium, a structure characterized by uniformly arranged stratified squamous cells interconnected by tight junctions, acts as a barrier against molecules larger than 10 Å^19^. The topical application of full-length immunoglobulins is considered poor due to their limited ability to penetrate the intact corneal epithelium. However, previous reports have shown that ocular inflammation disrupts the corneal epithelial function, allowing molecules such as bevacizumab, an immunoglobulin with a molecular weight of 149 kDa, to penetrate into the corneal stroma^20,21^. Similarly, we observe disruption of the corneal epithelium in our new model of LPS-induced keratitis, thus allowing HGF, a protein with a molecular weight of 84 kDa, to penetrate through the epithelium and into the corneal stroma.

HGF has been shown to suppress tissue scarring in various organs, including the lungs, heart, and skin^22–25^. In a murine model of mechanical injury, we have previously reported that the topical application of HGF prevents corneal scar formation^13^. Here, we demonstrate the capacity of HGF to suppress keratitis-induced corneal opacity formation to preserve corneal transparency, which is essential for optimal vision.

Fibrosis, a deleterious consequence of tissue repair, significantly impedes visual acuity and corneal function^26^. We and others have previously reported that HGF application prevents the generation of myofibroblasts following ocular and non-ocular tissue injury^13,14,27^. The stromal expression of α-SMA hallmarks the development of myofibroblast and corneal fibrosis^28,29^. In the present study, we observed increased expression of α-SMA following LPS injection which was significantly reduced following HGF treatment compared to PBS treatment, suggesting topical application of HGF suppresses myofibroblast formation in LPS-induced keratitis. However, a study by de Oliveira et al.^30^ reported no significant difference in the expression of α-SMA in HGF- and vehicle-treated rabbit corneas following PRK-induced injury. The discrepancy in findings could be attributed to the amount of HGF (0.1 mg/mL) used in de Oliveira et al. study^30^ which is 10-times lower than the doses (1 mg/mL) used in the present study and previous reports^13,14^.

The uniquely organized corneal structure is an integral factor for maintaining corneal strength and transparency^31^. Therefore, early intervention of microbial keratitis to preserve corneal tissue architecture is imperative for maintaining corneal clarity and normal function. Herein, we observed normalized tissue structure in HGF-treated mice, which were comparable to the naïve corneas and opposed to the PBS-treated corneas marked by a significant increase in corneal thickness. Specifically, HGF-treated corneas showed a significant increase in the stratification of the epithelial cell layer and reduction in stromal edema and thickness, further confirming the role of HGF in maintaining corneal cellular architecture following microbial keratitis.

Suppression of tissue inflammation following corneal infections is vital to preserving the corneal structure and accelerating tissue healing^32^. Thus, therapeutics with immunomodulatory properties ensure a more favorable outcome when used to treat microbial keratitis^33^. We observed that HGF, as an immunomodulatory molecule^10,34^, suppresses corneal inflammation and re-establishes immune quiescence following microbial keratitis, which is evidenced by decreased infiltration of immune cells and reduced expression of pro-inflammatory cytokine IL-1β in corneas treated with HGF. These findings corroborate our previous data of HGF mediated suppression of TNF-α and IL-1β expression in a dose-dependent manner following mechanical injury^10^.

In summary, our data derived from the new murine model of LPS-induced keratitis demonstrate that topical application of HGF prevents the progression of keratitis-induced corneal opacity and inflammation by suppressing α-SMA and pro-inflammatory cytokine expression and restoring corneal architecture. The present study highlights the anti-inflammatory and restorative function of HGF in keratitis, expanding the potential therapeutic application of HGF to infectious inflammatory ocular surface disorders.

## Materials and methods

### Animals

Six- to 8-week-old male C57BL/6 wild-type mice from Charles River Laboratories were used in the experiments. The protocol was approved by the Schepens Eye Research Institute Animal Care and Use Committee, and all animals were treated according to the Association for Research in Vision and Ophthalmology (ARVO) Statement for the Use of Animals in Ophthalmic Research and the Animal Research: Reporting of In Vivo Experiments (ARRIVE) guidelines.

### Induction of LPS keratitis

Mice were anesthetized with ketamine (200 mg/kg) and xylazine (10 mg/kg) (Patterson Veterinary Supply, Inc.) and 5 µg of ultrapure LPS isolated from *Escherichia coli* 0111:B4 (Invivogen) in 2 µl PBS were injected intrastromally to the central cornea of the right eye, as previously described^35^. Briefly, a small tunnel from the corneal epithelium to the anterior stroma was created using a 30-gauge needle (Hamilton Company, Reno, NV). Another 34-gauge needle attached to a 2.5-μL Hamilton syringe was passed through the tunnel into the stroma for the injection of LPS.

### HGF administration

To test the penetrance of HGF to the corneal epithelium, mice were topically treated with 3 µl of 0.1% His-tagged mouse recombinant HGF (His-Tag) (Sino Biological) following the second intrastromal injection of LPS. Intracorneal diffusion of HGF was confirmed with immunohistochemistry analysis using anti-his antibodies.

To evaluate the effect of HGF on LPS-induced corneal haze, mice were divided into two treatment groups: 0.1% murine recombinant HGF protein (R&D Systems). Mice were treated with 3 µl of HGF or control PBS, using a micropipette thrice daily for 5 days.

### Evaluation of corneal opacity

Corneal opacity was assessed by capturing brightfield images using a biomicroscope on day 4, 7, and 10 following the 2nd injection on day 4 (Fig. 2A)^36^. Images taken on day 4 and 10 were converted into binary mode, in which black areas correspond to areas of corneal opacity, and analyzed using NIH ImageJ software (version 1.34 s). Effect of HGF treatment in suppressing corneal opacity following induction of LPS keratitis was evaluated as percentage of restoration of corneal transparency calculated from day 4 on day 10 post-injection.

### Immunohistochemistry

Mice were sacrificed on day 10 and formalin-fixed paraffin-embedded (FFPE) sections (4 µm) of the whole eyeball were blocked with 2% BSA and anti-FcR antibodies (Affymetrix eBioscience). Cross-sections were then immunostained with Alexa Fluor 488-conjugated anti-His tag (Biolegend), anti-α-SMA (Affymetrix), or isotype control for overnight at 4 °C. Slides were then mounted using DAPI-containing VECTASHIELD® mounting medium (Vector Laboratories) and examined under a fluorescence microscope (Nikon Eclipse E800; Nikon Instruments, Melville, NY, USA). Expression of α-SMA was quantified by calculating percent of green pixels in the corneal section using NIH ImageJ software (version 1.34 s). Protein expression has been normalized to naive corneas to negate the non-specific autofluorescence reading.

### Histological analysis

Cross-sections were prepared from formalin-fixed whole eyeballs harvested on day 10 post-injection for hematoxylin and eosin (H&E) staining. Corneal tissue structure was analyzed under a bright-field microscope (Nikon Eclipse E800) at 20X magnification. Corneal thickness was measured using NIH ImageJ (version 1.34s) software.

### RNA isolation and real-time qPCR

Total RNA was isolated using the RNeasy Micro Kit (Qiagen), as previously described^37^. Isolated RNA was reverse transcribed into cDNA using oligo deoxy-thymidine (oligo (dT)) primer and SuperScript III First-Strand Synthesis System (Invitrogen). Real-time qPCR was then performed using Taqman Universal PCR Mastermix and Taqman primers for glyceraldehyde-phosphate dehydrogenase (*Gapdh*; Mm99999915_g1), Acta2 (*α-sma*; Mm01546133_m1), and IL-1β (Mm004324228_m1) (Life Technologies). The results were analyzed by comparative threshold cycle method and normalized to GAPDH as an internal control.

### Statistical analysis

Non-parametric Mann–Whitney tests were performed to determine significant mean difference between two treatment groups, set at *p* < 0.05. Data are presented as mean ± SEM. In vivo evaluations and quantification of images of corneal injury and opacity were performed in a masked fashion.
